# Photo-Induced Synthesis of Bioplastics from Xylan

**DOI:** 10.34133/research.1344

**Published:** 2026-07-08

**Authors:** Siyu Jia, Zixing Feng, Xueqing Yan, Zhiguo Zhang, Jingyan Zhu, Di Miao, Jun Rao, Zhengjun Shi, Junli Ren, Feng Peng

**Affiliations:** ^1^ MOE Engineering Research Center of Forestry Biomass Materials and Energy, School of Materials Science and Technology, Beijing Forestry University, Beijing 100083, China.; ^2^ Key Laboratory for Forest Resources Conservation and Utilization in the Southwest Mountains of China, Ministry of Education, Southwest Forestry University, Kunming 650224, China.; ^3^ State Key Laboratory of Pulp and Paper Engineering, South China University of Technology, Guangzhou 510641, China.; ^4^ State Key Laboratory of Efficient Production of Forest Resources, Beijing 100083, China.

## Abstract

Bioplastics derived from hemicellulose exhibit marked potential to substitute petroleum-based plastics due to their sustainability and biodegradability. However, developing bioplastics that combine facile manufacturing processes, excellent mechanical properties, and a low carbon footprint remains challenging. Herein, we present a strategy for fabricating high-performance bioplastics (XAGP) by crosslinking xylan molecular chains via photo-induced free-radical polymerization. The XAGP exhibits high light transmittance (95%), exceptional mechanical toughness (26 MJ/m^3^) and mechanical strength (84 MPa), outstanding thermal stability, water-assisted processability, rapid biodegradability (10 days in nature soil), hypotoxicity in aquatic environments, and good biocompatibility (cell viability above 98%). Furthermore, cost and life cycle assessment results demonstrate that XAGP is a more sustainable alternative to commercial biodegradable plastics, offering greater cost-effectiveness and a reduced carbon dioxide emission. This work establishes a promising route for transforming pulp waste into degradable, high-performance bioplastics, providing an effective solution to both waste disposal and plastic pollution.

## Introduction

Plastic serves as a cornerstone of modern society, playing an indispensable role in both daily life and industrial production. However, only 9% of plastic waste is recycled, while as much as 91% ends up in landfills or is incinerated [1]. Plastic pollution has become a serious global challenge. In particular, petroleum-based plastics undergo weathering and degradation in the natural environment, gradually forming micro- and nano-plastics, which exhibit remarkable environmental persistence [2–5]. Furthermore, plastic products contain tens of thousands of chemical additives, which can continuously migrate into the environment and biota throughout their life cycle, thereby posing additional health risks [6,7]. Despite the implementation of plastic bans and the promotion of recycling programs in numerous countries, the effectiveness of these measures remains limited due to low recycling rates and persistent reliance on fossil-based plastics [8,9]. Therefore, developing high-performance biomass-based biodegradable plastics is broadly accepted as the effective way of alleviating the aforementioned issues [10–12].

**Fig. 1.**
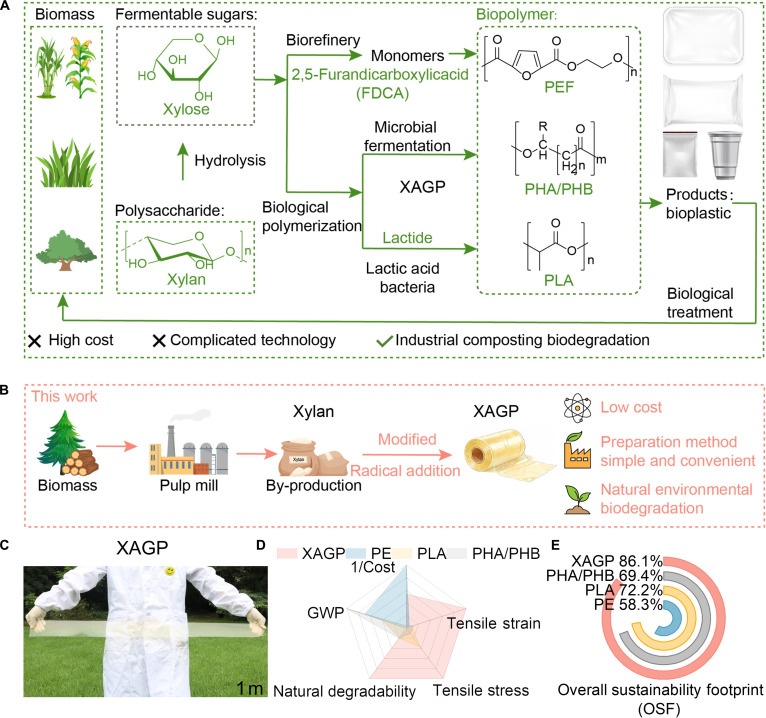
Synthesis routes from bio-based resources, and paper-mill waste to plastic. (A) Production of bioplastics from xylan via biorefining in a circular model. (B) Preparation of high-performance XAGP Bioplastic from xylan. (C) Image of XAGP. (D) Radar plot comparing XAGP with PE (petrochemical), PLA, and PHA/PHB (bio-based) plastics based on tensile stress, strain, 1/cost, GWP, and natural degradability. (E) Evaluating the overall sustainability footprint (OSF) of plastic materials.

In the past decade, extracting monomers from biomass via biorefining technology for synthesizing biodegradable plastics has emerged as a key strategy to replace petroleum-based plastics [13,14]. As the main degradation product of hemicellulose, xylooligosaccharides (XOS) serve as ideal feedstocks for producing functional monomers and demonstrate important potential in the field of bioplastics [15]. Through catalytic conversion, XOS can be efficiently transformed into high-value monomers including 2,5-furandicarboxylic acid (FDCA), fatty acid derivatives, and lactic acid (LA). Subsequently, these monomers undergo polymerization to form high-performance environmentally friendly plastics, including poly(ethylene 2,5-furandicarboxylate) [16,17], polyhydroxyalkanoates (PHAs)/poly-β-hydroxybutyrate (PHB) [18,19], and polylactic acid (PLA) [20,21]. However, the industrialization of bioplastics still faces several critical challenges: (a) From a production standpoint, their manufacturing relies on multistep chemical conversions and energy-intensive purification processes, resulting in high costs and a considerable global warming potential (GWP); (b) their biodegradability remains limited under nature environmental conditions, such as in soil and aquatic systems, and degrades effectively only in industrial composting facilities where temperatures exceed 60 °C (Fig. 1A).

To achieve low-cost, low-GWP, high-performance, and biodegradable bioplastics, the direct conversion of hemicellulose into bioplastic has garnered considerable interest. In the viscose fiber production process, large amounts of hemicellulose are generated as waste; after continuous alkaline treatment, the xylose content reaches as high as 96.6%, with a uniform structure and relatively high molecular weight, making it an ideal feedstock for sustainable materials. However, owing to this xylan-type hemicellulose highly ordered aggregated structure and pronounced intermolecular and intramolecular hydrogen bonding, xylan exhibits limited processability and solubility [22]. To develop xylan into a high-performance substitute for petroleum-based materials, chemical modification has emerged as an effective strategy to address its inherent limitations in processability and solubility. Studies have confirmed that strategies such as short alkyl chain modification [23–25], quaternization [26], carboxyalkylation [27], and dialdehyde functionalization [28] can markedly enhance the water solubility of hemicellulose. Nevertheless, water-soluble xylan derivatives still suffer from inferior film-forming capabilities and insufficient mechanical strength. Currently, enhancing the film-forming capacity and mechanical properties of xylan-based films is primarily achieved by incorporating polymers—such as cellulose nanofibers [29,30], polyvinyl chloride [31], and chitosan [32–34]—or inorganic fillers, such as montmorillonite hybrids [35] and graphene oxide [36]. However, because of limited energy dissipation systems and restricted molecular chain mobility, xylan-based bioplastic films struggle to meet the application requirements for both high strength and high toughness.

Herein, xylan-type hemicellulose, a by-product of the viscose industry, is employed as a sustainable feedstock and functionalized with unsaturated double bonds (XAG) via etherification, which allows for network formation via light-induced free-radical polymerization. Subsequently, controlled gradual dehydration combined with hot-pressing establishes an additional hydrogen-bonding network within the system, thereby fabricating a high-performance xylan plastic (XAGP). Remarkably, this material achieves an exceptional combination of high strength (84 MPa) and high toughness (26 MJ/m^3^) (Fig. 1B and C). Notably, the unsaturated double bonds introduced via molecular design enable efficient polymerization not only under 365-nm ultraviolet (UV) light but also upon exposure to natural sunlight, while the mechanical properties of the resulting XAGP remain unchanged. This characteristic substantially enhances the processability of the material. Compared with conventional nonbiodegradable polyethylene (PE) and commercial bioplastics such as PLA and PHA/PHB, XAGP offers a better combination in terms of mechanical properties (strength and toughness), cost-effectiveness, and environmental impact (Fig. 1D). With increasing emphasis on environmental sustainability, we evaluated the overall sustainability footprint (OSF) of XAGP, PE, PLA, and PHA/PHB plastic across 12 aspects from environmental, social, and techno-economic impacts (Fig. S1). Among these materials, XAGP achieved the highest OSF value (86.1%), indicating its superior sustainability performance (Fig. 1E). Furthermore, XAGP exhibits high transparency (95%), outstanding thermal stability, water-assisted processability, rapid degradability (complete within 10 days in natural environments), and excellent biocompatibility, offering a promising and competitive alternative to petroleum-based plastics.

## Results

### Preparation of XAGP

**Fig. 2.**
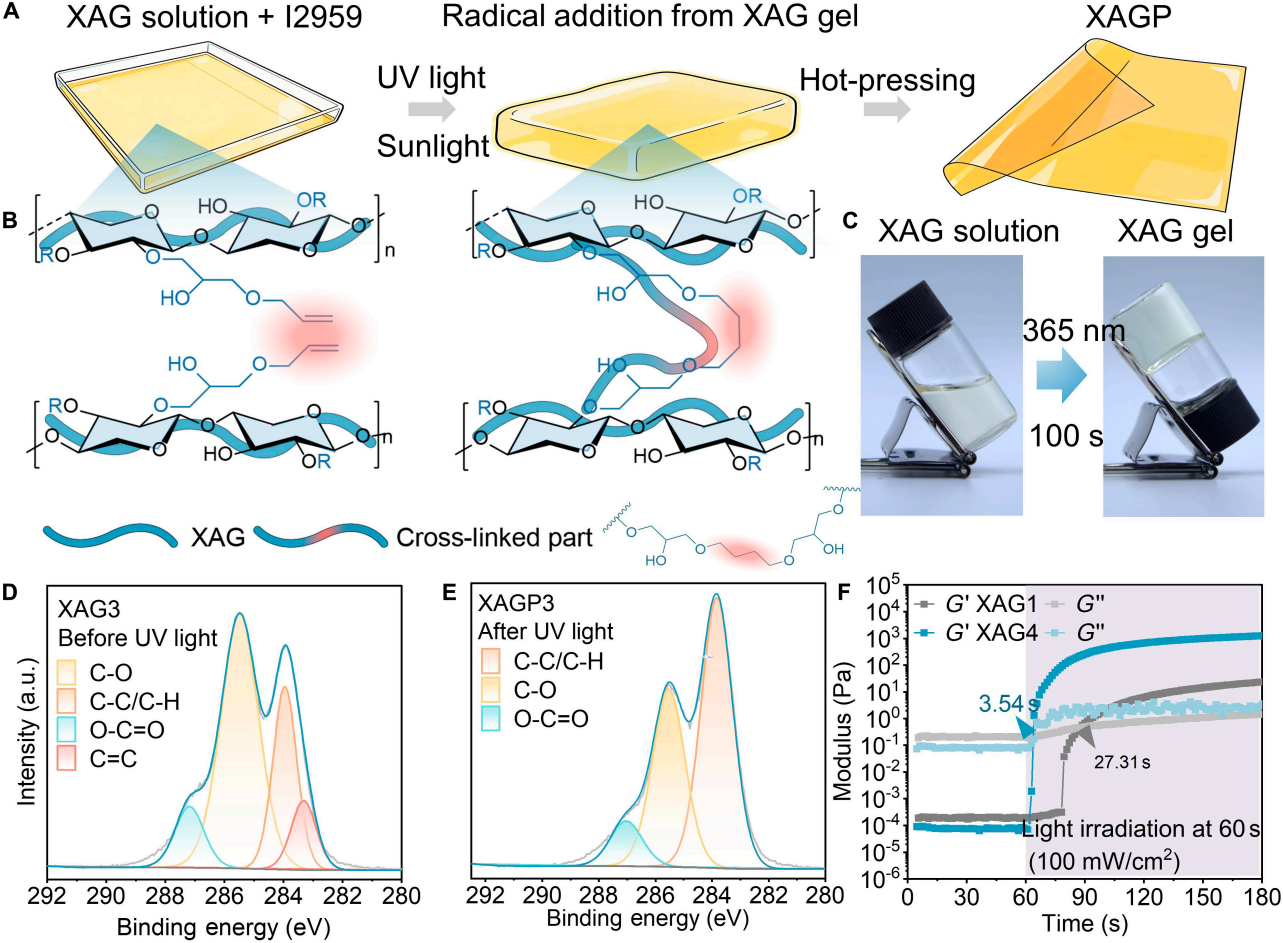
Radical polymerization of XAG. (A) Schematic of the fabrication of XAGP. (B) Schematic illustration on the UV-light-induced photo-crosslinking from a transparent XAG solution to XAG hydrogel. (C) Photo of the transition of solution to gel under UV light. XPS spectra of (D) XAG3 and (E) XAGP3. (F) Rheological monitoring of photo-crosslinking in XAG at different ratios.

The preparation process of XAGP is illustrated in Fig. 2A. Water-soluble XAG was synthesized from xylan via base-catalyzed etherification (Fig. S2A). A comparison of the Fourier transform infrared (FT-IR) spectra of Xylan and XAG (Fig. S2B) reveals a shift of the peaks of O–H to a higher wavenumber in XAG (from 3,425 to 3,445 cm^−1^), due to the difference between the new -OH bonds in allyl glycidyl ether (AGE) and -OH bonds in xylan [37]. The characteristic C=C stretching vibration of AGE (at approximately 1,641 cm^−1^) overlaps with the water adsorption peak of xylan (around 1,640 cm^−1^), making them difficult to distinguish. Hence, the chemical structure of XAG was further characterized by ^1^H nuclear magnetic resonance (NMR) and ^13^C NMR spectroscopy. In ^1^H NM spectra (Fig. S2C), the newly emerged signal at 5.8 parts per million (ppm) (H8) along with the split peaks at 5.0 ppm (H9) are attributed to unsaturated vinylene protons. The peak observed at 4.0 ppm is assigned to the methylene protons adjacent to the vinyl group (H6 and H7) [38–40]. In ^13^C NMR spectra (Fig. S2D), the new signals observed at 115.7 ppm (C8) and 135.4 ppm (C9) are attributed to the vinyl groups in XAG [38–40]. These results indicate the etherification success between the -OH of xylan and epoxy groups of AGE. The x-ray diffraction (XRD) patterns of xylan and XAG are presented in Fig. S2E. The characteristic diffraction peaks appearing at 11.5° (020), 12.8° (021), 19.6° (200), 23.3° (202), 25.8° (042), and 32.0° (025) demonstrated the crystalline structure of xylan. The XRD pattern of XAG exhibits a broad peak at 2*θ* = 19.6°, indicating that the introduction of AGE disrupts intermolecular hydrogen bonding and consequently inhibits the recrystallization of xylan. XAG exhibited a much higher maximum degradation temperature (*T*_max_) than xylan, which is attributed to the successful grafting of AGE onto xylan (Fig. S2F and G). The degree of substitution (DS) and double-bond content of XAG were determined from ^1^H NMR spectra (Fig. S2C). With the increase in the molar ratio of AGE, both the DS and the double bond content of XAG increased (Fig. S3A and Table S1). Meanwhile, the solubility of XAG varied with increasing DS (Fig. S4). After etherification modification, the weight-average molecular weight (Mw) of XAG increased from 52,200 to 65,514 g/mol (Fig. S3B).

The introduction of unsaturated C=C double bonds into the xylan via etherification modification not only markedly enhanced its water solubility but also provided active sites for subsequent free-radical copolymerization (Fig. 2B). The water-soluble photoinitiator (I2959) was selected to initiate free-radical polymerization under 365-nm UV irradiation, resulting in the rapid transition of the initially transparent XAG solution into a transparent gel (denoted as XAG gel) (Fig. 2C and Fig. S4). The occurrence of free-radical copolymerization in XAG was confirmed by x-ray photoelectron spectroscopy (XPS), as shown in Fig. 2D and E. The C1s spectrum of XAG before photopolymerization exhibited a peak at a binding energy of 283.3 eV, which was assigned to C=C bonds. However, after photopolymerization, the C=C peak disappeared, indicating that the unsaturated C=C double bonds in XAG were completely consumed during the radical copolymerization process. The crosslinking density of XAG gels with different double-bond content was determined using low-field ^1^H NMR (LF ^1^H NMR) spectroscopy. Compared to XAG1 gel, the transverse relaxation time (*T*_2_) of XAG4 gel exhibited a marked reduction (Fig. S5), indicating a higher crosslinking density. This effect can be attributed to the increased number of double bonds in XAG4, which provide more active sites for free-radical copolymerization. Photorheological analysis was conducted to evaluate the radical reactivity and photo-crosslinking kinetics of XAG samples with different double-bond contents. As shown in Fig. 2F, crosslinking of XAG1 and XAG4 occurred after 27.31 and 3.54 s of UV irradiation at 100 mW/cm^2^, respectively. The photo-crosslinking process yielded maximum storage moduli (*G*′) of 24.04 kPa for XAG4 and 1.19 Pa for XAG1 within 100 s, ultimately leading to the formation of XAG gels. This confirms that the double-bond content of XAG critically governs the photopolymerization rate and markedly enhances the crosslinking degree. Furthermore, when the light intensity was reduced from 100 to 20 mW/cm^2^, the gelation time of XAG4 correspondingly increased from 3.54 to 8.44 s (Fig. S6). This result demonstrates that the high reactivity of the double bonds in XAG facilitates robust photo-crosslinking, even under reduced light intensity.

**Fig. 3.**
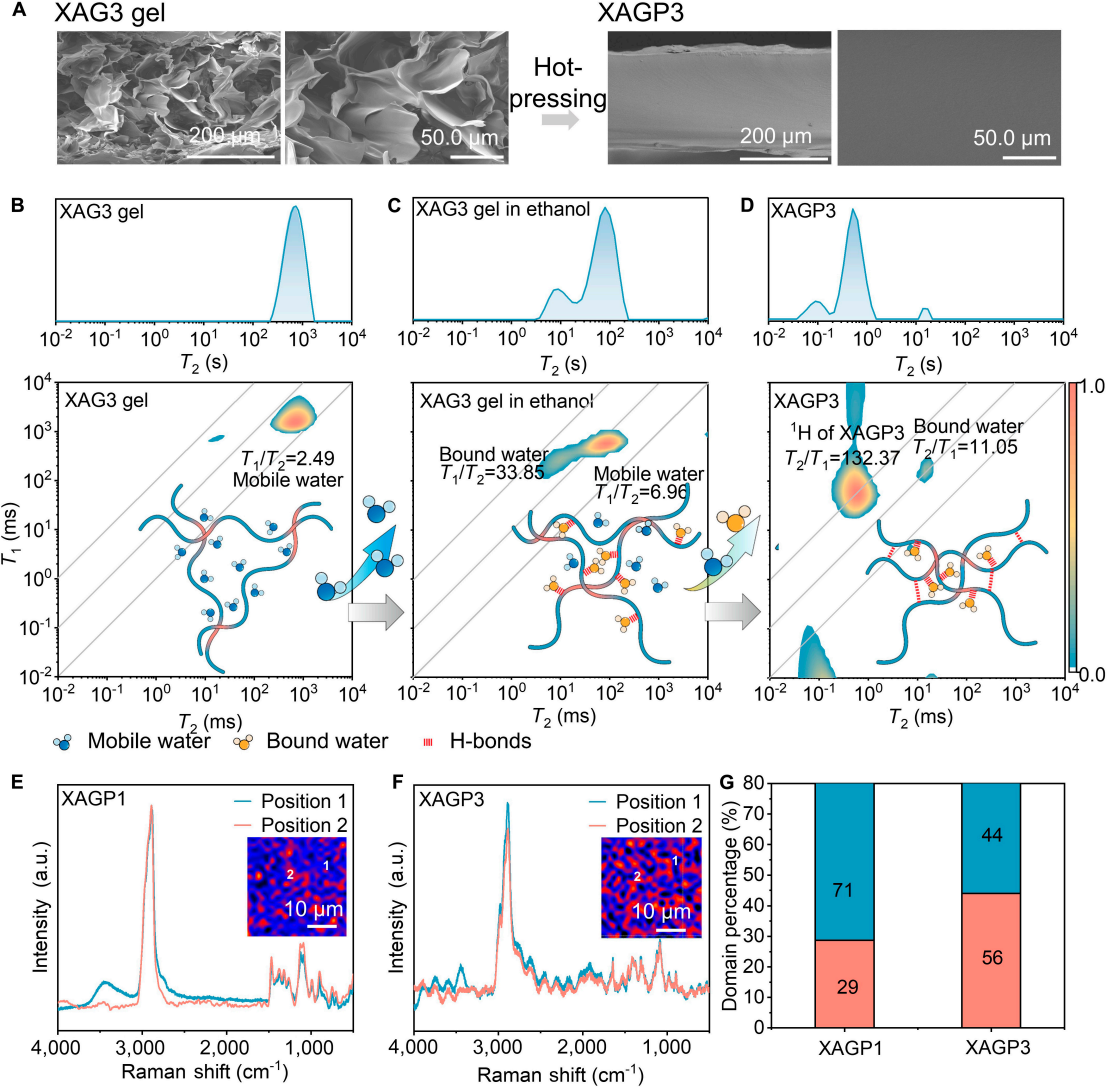
The microstructure of XAGP. (A) SEM images of the cross-sections of XAG3 gel and XAGP3. 2D LF ^1^H NMR spectra of (B) XAG3 gel, (C) XAG3 gel in ethanol, and (D) XAGP3 after hot press. (E and F) Raman spectra of positions 1 (blue) and 2 (red) within XAGP1 and XAGP3. The area ratio Raman image (30 μm × 30 μm) of XAGP1 and XAGP3 (insideinset picture). (G) The percentage of physical crosslinking (blue) and chemical crosslinking (red) domains within the XAGP samples.

During the fabrication of XAGP from XAG gel, a dense hydrogen-bond crosslinked network was progressively established among the molecular chains. This structure is formed through the synergistic action of gradient ethanol dehydration and subsequent hot-pressing. Scanning electron microscopy (SEM) analysis showed that the network structure of the original XAG3 gel was densified, resulting in the formation of XAGP3 with a uniform structure (Fig. 3A). During the dehydration process, the noncovalent interaction network effectively enhanced intermolecular forces, thereby considerably improving the mechanical properties of XAGP. The evolution of longitudinal relaxation (*T*_1_) and *T*_2_ constants in 2D-LF ^1^H NMR spectroscopy was analyzed to elucidate the mechanism of hydrogen-bond network formation among XAG molecules [41,42]. The *T*_1_/*T*_2_ ratio serves as a critical indicator of water molecular dynamics, exhibiting an inverse correlation with the extent of molecular motional freedom. Generally, a lower *T*_1_/*T*_2_ ratio indicates higher molecular mobility, whereas a diagonal line where *T*_1_/*T*_2_ = 1 corresponds to the state of a highly mobile liquid [41]. As shown in Fig. 3B and Fig. S7A, XAG gel exhibits a *T*_2_ peak at 728 ms, with a corresponding *T*_1_/*T*_2_ ratio of 2.49. These results suggest the absence of a densely crosslinked network capable of effectively restricting water molecular mobility within the gel. Following ethanol gradient dehydration, the *T*_2_ peaks shifted upfield, resulting in 2 distinct peaks at 81 and 8 ms. Concurrently, the *T*_1_/*T*_2_ ratio increased to 2 distinct values of 6.96 (for free water) and 33.8 (for bound water). These changes reflect the progressive removal of free water from the system, accompanied by strengthened intermolecular hydrogen bonding between XAG and water molecules, which facilitates the formation of bound water (Fig. 3C and Fig. S7B). After hot-pressing, the characteristic peak of free water in XAGP disappeared, while the water molecular peak continued to shift upfield, forming peaks at 0.517 and 15.69 ms. Additionally, the *T*_1_/*T*_2_ ratios of 132.37 (for H protons) and 11.05 (for bound water) further demonstrate the formation of a highly dense hydrogen-bonded network among the polymer chains (Fig. 3D and Fig. S7C).

To further elucidate the spatial distribution characteristics of the local chemical composition in XAGP, confocal Raman spectroscopic imaging was employed to analyze the XAGP1 and XAGP3. The characteristic peaks at 3,500 to 3,000 cm^−1^ and 3,000 to 2,750 cm^−1^ correspond to the stretching vibrations of the –OH groups and the C–H bonds in –CH_2_ groups, respectively [43,44]. Based on the distribution of the –OH/–CH_2_ ratio, the red regions (lower ratio) represent chemically crosslinked domains, whereas the blue regions (higher ratio) correspond to physically crosslinked domains. By comparing the relative distributions of these 2 regions, it can be observed that the hydroxyl content in XAGPs samples exhibits pronounced heterogeneity at the microscale. Furthermore, point “1” was selected from the blue region and point “2” was selected from the red region to extract their corresponding one-dimensional Raman spectra. The results show that the intensity of the hydroxyl characteristic peak (3,441 cm^−1^) in the blue region is markedly higher than that in the red region, indicating that the blue region is a hydroxyl-rich domain dominated by physical crosslinking, whereas the red region is a hydroxyl-deficient domain dominated by chemical crosslinking (Fig. 3E and F). It should be noted that, due to the strong fluorescence interference in XAGP3, the quality of its one-dimensional Raman spectra is relatively poor. By comparing the relative area fractions of the blue regions, it is evident that the proportion of physically crosslinked domains in XAGP1 (71%) is higher than that in XAGP3 (44%), indicating that XAGP1 contains a greater extent of physical crosslinking structures (Fig. 3G). It may be attributed to the relatively loose network structure of the XAG1 gel, which facilitates rapid shrinkage during dehydration, thereby promoting the formation of more hydrogen bonding interactions [45]. However, excessive physical crosslinking can render the XAGP1 more brittle, thereby compromising the structural integrity of the sample.

### Performance of XAGP

**Fig. 4.**
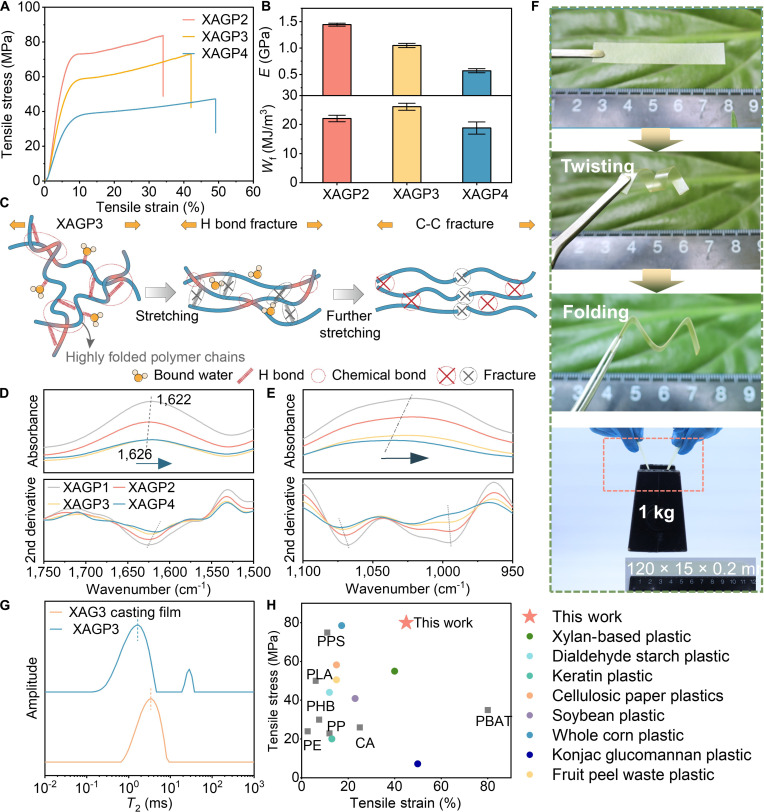
Mechanical properties of XAGP. (A) Stress–strain curves, (B) toughness (*W*_f_) and Young’s modulus (*E*) values of XAGP with different ratios. (C) Schematic illustration of the fracture mechanism of the XAGP. (D and E) Contrast FT-IR spectra of different ratios of XAGP as well as their corresponding second-derivative plots. (F) Digital photograph of an XAGP strip (120 × 15 × 0.2 mm) demonstrates its flexibility and strength. (G) LF ^1^H NMR spectra of XAGP3 and XAG3 casting film. (H) Tensile stress and tensile strain of XAGP compared with currently widely used plastics (cellulose acetate [CA]; polyethylene [PE]; polypropylene [PP]; polyphenylene sulfide [PPS]; polyhydroxybutyrate [PHB]; polylactic acid [PLA]; poly(butylene adipate-terephthalate) [PBAT]) and other bio-based plastics.

As the double bond content and molecular weight increased, the *T*_2_ peak of the resulting XAGP shifted upfield, indicating a higher crosslinking density (Fig. S8). Consequently, both the mechanical strength and Young’s modulus (*E*) of XAGP gradually decreased, from 83.7 to 45.2 MPa and from 1.4 to 0.6 GPa, respectively (Fig. 4A and B). However, from XAGP2 to XAGP4, the toughness (*W*_f_) initially increased and then subsequently decreased, with *W*_f_ reaching a maximum value of 26 MJ/m^3^. As shown in Fig. 4C, the XAGP network exhibits notable mechanical dissipation behavior, which can be divided into 3 stages: Initially, during the early stretching phase, the highly folded XAG molecular chains undergo extension; subsequently, as the strain increases, hydrogen bonds within the network preferentially break, while the migration of water molecules functions as plasticizer; ultimately, at the maximum strain, the chemical crosslinking points progressively fracture, and XAGP completely fractures. The higher molecular weight of XAG4 promotes the formation of complex chain entanglements, which necessitates greater energy dissipation in the early stretching phase, thereby resulting in reduced mechanical strength but enhanced fracture elongation. To gain insight into the molecular interactions that underlie the mechanical properties of XAGP, we conducted FT-IR spectroscopy (Fig. 4D and E). In this work, we compared the C=O stretching [*v*(C=O)] regions (1,750 to 1,500 cm^−1^) and the C–O stretching [*v*(C–O)] region (1,100 to 950 cm^−1^), to explain the molecular interactions of the XAGP with different crosslinking density networks. To enhance spectral resolution, second-derivative curves were generated, where the sharpened minima correspond to the absorption maxima in the original spectra [46,47]. For the *v*(C=O) band, a blue shift of the main peak at 1,626 cm^−1^ was observed as the crosslinking density decreased, indicating enhanced dehydration of C=O groups (Fig. 4D). Within the ν(C–O) region, the peak around 1,090 cm^−1^ originates from the C−O−C stretching vibration along XAG chains. The band centered approximately at 1,000 cm^−1^ corresponds to C–O–C groups engaged in strong hydrogen bonding interactions within the XAGP network. With decreasing crosslinking density, the shoulder peaks exhibited blue shifts, indicating the formation of stronger hydrogen bonds (Fig. 4E). These changes indicate that enhanced dehydration of C=O and C–O–C groups (driven by decreased crosslinking density in XAGP) promoted the formation of a greater number of hydrogen bonds, thereby improving tensile stress and Young’s modulus. Specifically, during the preparation of XAGP1, the sample became highly brittle after hot-pressing owing to its low crosslinking density, which precluded mechanical property testing. As illustrated in Fig. 4F, XAGP3 exhibits high flexibility, allowing it to be twisted and folded freely, while also supporting a weight of 1 kg without fracture.

Furthermore, to demonstrate the reinforcing effect of the crosslinked network on the mechanical properties of XAGP, a comparative analysis of mechanical performance was conducted between XAGP and XAG casting films (Fig. S9). XAGP exhibits superior mechanical stress and substantially enhanced fracture strain. Consistent with this, LF ^1^H NMR spectroscopy revealed an upfield shift in the signal peak for XAGP3 compared to that of the XAG3 casting film. This shift indicates stronger confinement of protons within the crosslinked network of XAGP, thereby corroborating its energy-dissipative structural characteristics. Moreover, the presence of a bound water characteristic peak in the XAGP3 LF ^1^H NMR spectrum further supports the plasticizing effect of water molecules on XAGP (Fig. 4G).

As shown in Fig. 4H and Table S2, XAGP developed in this work demonstrates a combination of high strength (84 MPa) and large fracture strain (52%)—surpassing many petroleum-based and bio-based plastics—attributable to its strategically designed dissipative network. Notably, XAGP demonstrates a high toughness that is markedly higher than that of previously reported xylan-based materials [28,48,49]. Generally, strength and toughness are mutually exclusive in most bio-based plastic. With superior mechanical properties, XAGP provides a potential alternative to traditional packaging films in sustainable applications.

**Fig. 5.**
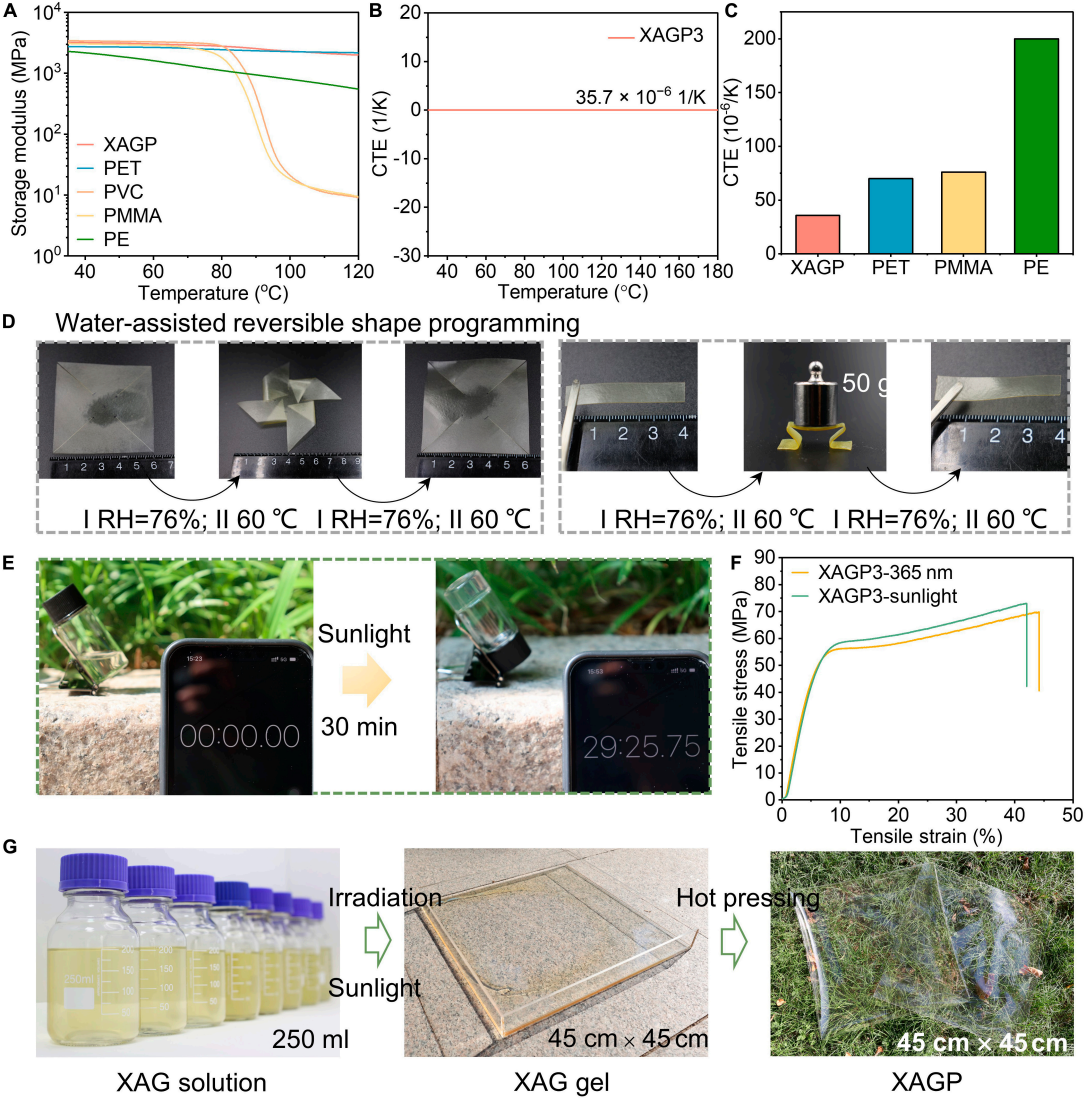
Thermal stability, water plasticity, and sunlight-driven preparation. (A) DMA results comparing the storage modulus of XAGP3 with that of common polymers (PET, PVC, PMMA, and PE). (B) Analysis of dimensional changes in XAGP3 and (C) comparison of its CTE against with PET, PMMA, and PE. (D) Schematic diagram of the forming process of the water-assisted reversible hydroplastic XAGP. (E) A photograph showing the transition of an XAG3 solution into a gel under natural sunlight at 35 °C in Beijing. (F) Stress–strain curves comparing XAGP3-365 nm with XAGP3-sunlight. (G) Mass-produced XAGP under sunlight. Images of the scaling production of XAG solution, XAG gel, and XAGP.

As a potential alternative to replace petroleum-based plastics, XAGP possesses outstanding optical properties, with a high degree of transparency (95%), as shown in Fig. S10. It also exhibits selective solvent resistance, remaining stable in low-polarity solvents (e.g., ethanol and tetrahydrofuran), while undergoing swelling behavior in high-polarity solvents (e.g., water and dimethyl sulfoxide) (Fig. S11). Furthermore, with increasing crosslinking density, the water contact angle of XAGP rises from 65° to 94°, reflecting enhanced hydrophobicity (Fig. S12). For plastic materials, maintaining dimensional and shape stability at elevated temperatures is crucial. Dynamic mechanical analysis (DMA) reveals that XAGP3 exhibits excellent performance stability over the temperature range of 30 to 120 °C. Even at 120 °C, it retains a storage modulus of 2.3 GPa, comparable to that of high-stability PET (Fig. 5A). Moreover, XAGP3 possesses a relatively low coefficient of thermal expansion (CTE), measured as 35.7 × 10^−6^/K at 180 °C (Fig. 5B). In comparison with the CTE values of commonly used commercial plastics [50], XAGP demonstrates outstanding shape stability under high-temperature conditions (Fig. 5C). XAGP also exhibits unique hydroplastic behavior. After exposure to 76% relative humidity (RH) for 30 min, it absorbs approximately 6 wt% moisture. The absorbed water enables the bioplastic to be folded into designed shapes. Subsequent drying at 60 °C removes the water and fixes the configured form. When placed again in 76% RH for 30 min, XAGP3 recovers its initial state, allowing reprocessing. When the material was folded into a chair-like structure and dried, the resulting structure could support a load of approximately 50 g (Fig. 5D). These properties demonstrate that XAGP is amenable to water-assisted programming, enabling the creation of high-strength and reshapable structures with versatile morphologies.

To save costs and facilitate large-scale production, we verified the possibility of XAG polymerizing under sunlight. Figure 5E demonstrates that the XAG solution transitions into crosslinked XAG gel within 30 min under sunlight illumination due to its high reactivity. Mechanical performance tests revealed that XAGP3 prepared under both sunlight and 365-nm UV light exhibited highly consistent mechanical properties (Fig. 5F and Fig. S13). Therefore, XAGP can be prepared under natural illumination conditions, substantially reducing energy demands and enabling scalable production (Fig. 5G).

### Environmental friendliness, techno-economic, and life cycle assessment of XAGP

**Fig. 6.**
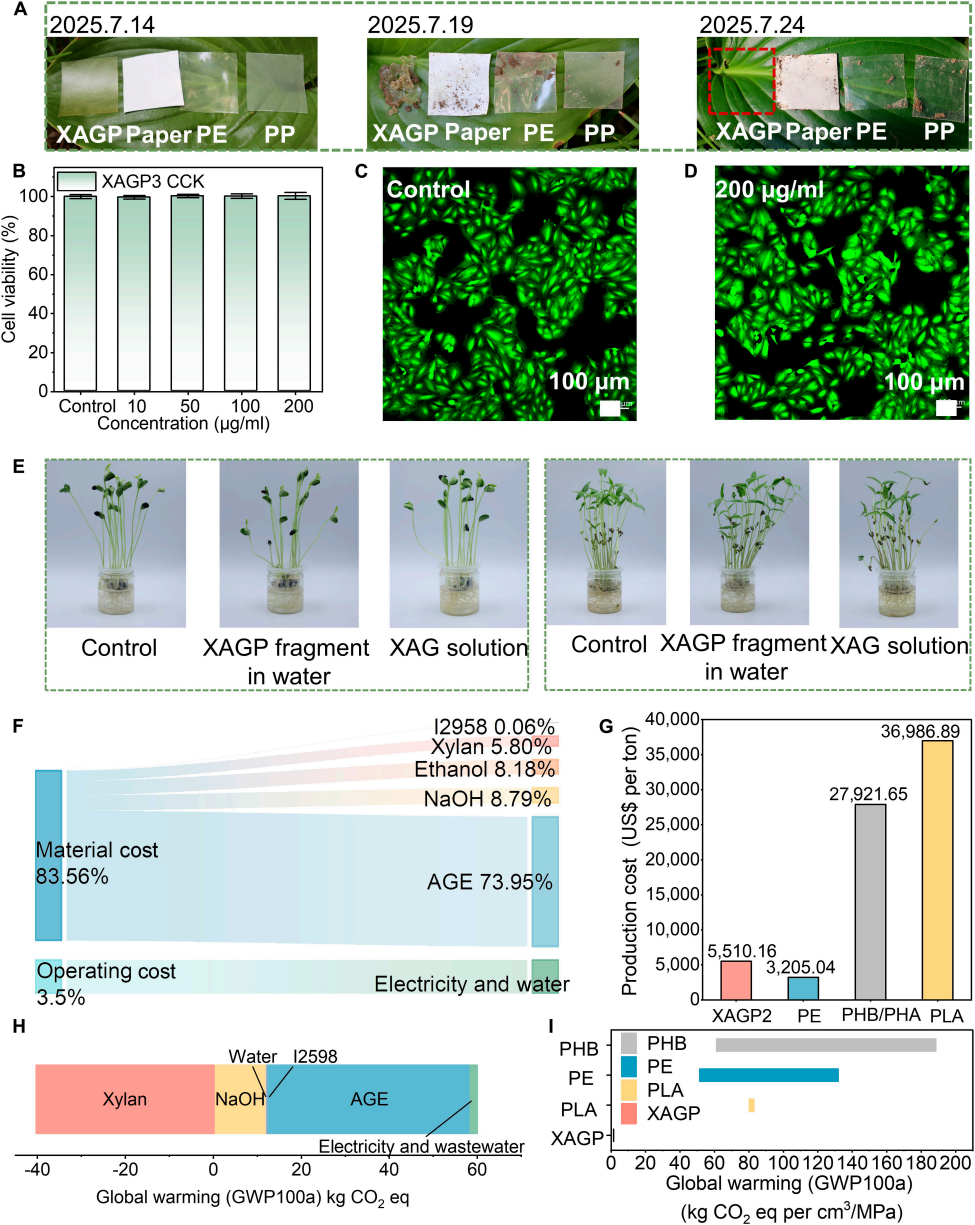
Environmental impacts of XAGP. (A) The biodegradation process of XAGP, filter paper, PE film, and PP film in the soil. (B) Cytotoxicity of XAGP3 was evaluated in vitro using the CCK-8 assay after 24 h of exposure in culture media containing different concentrations of the material. Live/dead staining with acridine orange (AO) and ethidium bromide (EB) was performed on mouse fibroblast cells (A549) cultured for 24 h: (C and D) control group, and group treated with 200 μg/ml [the sample was tested based on ISO 10993–5:2009 (E)]. (E) Plant growth and environmental impacts of XAGP fragment and XAG solution (growth period: 5 days). (F) Detailed production costs of XAGP in US$. (G) The production cost (US$) of XAGP compared to PE, PLA, and PHA/PHB film (1t product). (H) Global warming potential (GWP100a) breakdown values for XAGP production. (I) GWP of XAGP compared to PE, PLA, and PHA/PHB plastic (per cm^3^/MPa).

To prove the concept of biodegradability of XAGP, we buried XAGP, paper, PE, and PP plastic in natural soil on the campus of Beijing Forestry University. As shown in Fig. 6A, XAGP can be rapidly degraded within 10 days, which is attributed to the capacity of bacteria and fungi to directly decompose the xylan molecules, coupled with the presence of moisture in natural soil. Compared with the fossil-based PE plastic, and bio-based PLA and PHA/PHB plastic, XAGP demonstrates a marked advantage in biodegradability (Table S3). Biosafety is a critical factor for the practical application of XAGP in daily-use plastic. The biocompatibility of XAGP was assessed using the Cell Counting Kit-8 (CCK-8) assay. As shown in Fig. 6B, cell viability remained above 98% after 24 h of culture with different concentrations of XAGP used as substrate. This result indicates that XAGP exhibits no cytotoxic effects. Confocal laser scanning microscopy was employed to assess cell viability by visualizing live/dead cell distributions at concentrations of 0 and 200 μg/ml (red regions indicate the presence of dead cells) (Fig. 6C and D). XAGP exhibited excellent cell viability at both 0 and 200 μg/ml concentrations. The excellent biocompatibility of XAGP offers broad prospects for its application in packaging materials. To further evaluate the ecological safety of XAGP in aquatic environments, a hydroponic experiment was conducted using mung beans (*Vigna radiata*) and black beans (*Glycine max*) as model organisms for toxicological evaluation. The experiment included 3 treatment groups: (a) a blank control group using deionized water, (b) aqueous treatment containing XAGP fragments, and (c) XAG solution treatment. By comparing the growth responses of leguminous plants across different treatment groups, the environmentally friendly characteristics of XAGP were demonstrated (Fig. 6E).

Based on a comprehensive analysis of raw material procurement costs and production processes for XAGP, this study comprehensively evaluates its production cost, thereby demonstrating the commercial feasibility of large-scale production (Table S4). The cost assessment reveals that the raw material cost of AGE accounts 73.95% of the total production cost in the fabrication process of XAGP (Fig. 6F). Hence, the main strategy for future cost optimization should focus on reducing chemical feedstock expenditures. Compared to PHA/PHB and PLA bioplastic, XAGP demonstrates markedly lower manufacturing costs (Fig. 6G and Table S5). The system boundary flow diagram for manufacturing XAGP, which includes the source of raw materials, processing, and waste treatment, is shown in Fig. S14. The environmental impact of XAGP production was evaluated using life cycle assessment methodology (Fig. S15 and Table S6). The environmental impact potentials were quantified using the CML-IA method, including the following categories: Abiotic depletion (ADP-elements), Abiotic depletion (fossil fuels) (ADP fossil), Global warming potential (GWP 100a), Ozone layer depletion (ODP), Human toxicity (HTP), Freshwater aquatic ecotoxicity (FAETP), Marine aquatic ecotoxicity (MAETP), Terrestrial ecotoxicity potential (TEP), Photochemical oxidation (POCP), Acidification (AP), and Eutrophication (EP) (Fig. S16 and Table S7). The data for PE and PLA were obtained from the Ecoinvent v3 database, while the data for PHB were sourced from the literature. In this study, the raw material was derived from waste from the paper industry. Conventional disposal methods typically involve combustion as fuel, which releases substantial amounts of CO₂. After being processed into XAGP, the material enables long-term carbon sequestration, thereby reducing environmental impact. In addition, the carbon sequestration effect of xylan systematically counterbalances the environmental impact associated with chemicals used in prepare XAGP. In particular, the GWP of XAGP is partially offset (–40.2%) by the carbon-negative effect of xylan. However, AGE (46.1%) and NaOH (12.2%) remain the main contributors to its environmental burden (Fig. 6H). In addition, the environmental impact of XAGP was evaluated against PE, PLA, and PHB plastics using a normalization approach based on material density and tensile strength (Fig. 6I and Fig. S16, and Tables S8 and S9). The data used in evaluation are obtained from the Ecoinvent v3. Overall, XAGP exhibits minimal environmental impact (ADP: 3.60E−05; ADP [fossil fuels]: 8.41E+01; GWP: 1.46; ODP: 1.67E−07; HTP: −5.84; FAETP: −1.11; MAETP: 1.79E+03; TEP: −1.12; POCP: 2.45E−04; AP: 1.02E−02; EP: 4.64E−03), and its manufacturing process represents a sustainable and environmentally friendly pathway.

## Conclusion

In summary, this work introduces a sustainable bioplastic fabricated from paper-mill waste via light-mediated polymerization and hot-pressing. The double bond in XAG exhibits high reactivity toward free-radical polymerization, enabling XAGP to be fabricated under either UV light or natural sunlight. By undergoing gradual dehydration followed by hot-pressing, XAGP develops a dissipative network structure that endows it with impressive mechanical properties: a high strength of 84 MPa and exceptional toughness reaching 26 MJ/m^3^. These values surpass those of most petroleum-based plastics, commercially available biodegradable polymers, and previously reported biomass-derived films. Meanwhile, the XAGP also demonstrates high transparency, exceptional thermal stability, water-assisted processability, rapid biodegradability in all-natural environments, low toxicity, good biocompatibility, and convenient manufacturing. Compared with PE, PLA, and PHA/PHB plastic, XAGP exhibits superior cost-effectiveness and a reduced environmental impact throughout their life cycle. Overall, this work establishes a viable strategy for producing sustainable and eco-friendly alternatives to petroleum-based plastics.

## Materials and Methods

### Materials

The xylan used in this study was sourced from hardwood pulp (Yibin Grace Group Co., Ltd.) following our previous work [28]. Sugar composition analysis revealed that the xylan consisted of xylose (96.6%), glucuronic acid (0.5%), galactose (0.2%), and glucose (2.7%) in relative molar percentages. Gel permeation chromatography (GPC) analysis determined the molecular weight characteristics of the pristine xylan, showing an Mw of 38,050 g/mol, a number-average molecular weight of 22,590 g/mol, and a polydispersity index of 1.68. AGE, sodium hydroxide (NaOH), and 2-hydroxy-4′-(2-hydroxyethoxy)-2-methylpropiophenone (I2958) were obtained from Shanghai Macklin Chemical Co., China.

### Synthesis of XAG

For preparing the XAG, xylan power (26.4 g) was dissolved in 5 wt% NaOH aqueous solution (400 ml) at 50 °C to form a transparent solution. After dissolution, the xylan solution is cooled to room temperature. Subsequently, AGE was added dropwise into the stirred xylan solution until the desired molar ratios of AGE to xylan (1:1, 1:2, 1:3, and 1:4) were achieved.

Subsequently, AGE was added dropwise into the stirred xylan solution until the desired molar ratios of hydroxyl groups of the xylose units to epoxy groups of AGE (1:1, 1:2, 1:3, and 1:4) were achieved. The reaction was then allowed to proceed under continuous stirring at 30 °C for 24 h. After reaction completion, the pH of the system was neutralized with diluted HCl. The resulting solution was purified by dialysis using a membrane with a molecular weight cutoff of 6,000 to 8,000 Da for 1 week to remove salts formed during neutralization and unreacted AGE. The modified xylan is recorded as XAG-*x* (*x* represents the molar ratios of AGE). To investigate the chemical characteristics, XAG-*x* powder was obtained by freeze-drying.

### Preparation of XAGP

Take the preparation of XAGP3 as an example. The XAG3 that was after dialysis was concentrated to 5 wt% by rotary evaporation. A measured quantity of XAG3 solution was mixed with 0.1 wt% I2958 (photoinitiator) until complete dissolution. The homogeneous solution was transferred into a mold, and initiated under UV light (*λ* = 365 nm, intensity = 30 mW/cm^2^ for 5 min) to form the XAG3 gel. The XAG3 gel was immersed into the anhydrous alcohol for dehydration. Subsequently, the XAG3 gel was processed into transparent film through hot-pressing (80 °C, 20 MPa, 3 h), with the resulting material termed XAGP3.

### Structure characterization

FT-IR spectra of xylan, AGE, XAG, and XAGP were recorded in a Thermo Fisher Nicolet iS5 equipment, and the wavenumber ranged from 4,000 to 500 cm^−1^. The XRD patterns were obtained using an x-ray diffraction analyzer (D8 Advance, Bruker) with Cu Kα radiation (*λ* = 0.154 nm) operated at 36 kV and 20 mA. The measurements were performed in the 2*θ* range of 10° to 60° with a scanning rate of 2°/min. The sample morphologies were characterized by a scanning electron microscope (Regulus 8100) at 5 kV. The molecular weight distribution of the samples was analyzed by GPC (LC-20A, Shimadzu, Japan). The thermal stability of the samples was evaluated using a thermogravimetric analyzer (TG 209 F3 Tarsus) under nitrogen atmosphere. The measurement was conducted from room temperature to 600 °C with a heating rate of 10 °C/min. XPS spectra were obtained from a Thermo Scientific K-Alpha X-Ray Photoelectron Spectrometer System. Charge correction was performed in Advantage software by referencing to the C–C peak at 284.6 eV. LF ^1^H NMR spectra were recorded on a Niumag VTMR20-010V-I analyzer, with samples sealed in glass vials to prevent water loss. The 2D LF ^1^H NMR *T*_1_−*T*_2_ spectra and transverse relaxation (*T*_2_) curves were obtained using an SR-CPMG and CPMG pulse sequence. The rheology properties of the XAG were measured by an HAAKE Viscotester iQ using a parallel plate geometry (25 mm diameter) at 25 °C. The photo-crosslinking kinetics of XAG were determined underusing shear rheological test with a gap distance of 0.1 mm at a constant angular frequency and strain of 10 rad/s and 0.5%, respectively. The samples were presheared at 100 s^−1^ for 60 s, and then irradiated for 180 s by a light source (320 to 500 nm) with different light densities through a transparent quartz bottom. The Raman map was collected using a spatial resolution of 1.3 μm, and the excitation laser wavelength was 532 nm (scan range: 30 μm × 30 μm). The tensile properties of the samples were evaluated using a universal testing machine (Instron 5982) equipped with a 1-kN load cell. Specimens were cut into rectangular strips (15 mm × 2 mm) and stretched at a constant crosshead speed of 2 mm/min. Prior to testing, the samples were conditioned in a controlled-temperature and humidity environment for 72 h to ensure equilibrium. To ensure statistical reliability, at least 5 replicates were tested, and the average values were reported. The toughness (*W*_f_) and elastic modulus (*E*) were derived from the initial linear region of the stress–strain curves. The optical properties of the samples were evaluated using a UV–vis spectrophotometer (PERSEE T9) equipped with an integrating sphere. Measurements were performed across a wavelength range of 200 to 800 nm. Water contact angle measurements were performed at room temperature using an SCI500M goniometer. The plastic samples and XAGP were tested using DMA NETZSCH 242E Artemis under N_2_ atmosphere, with a temperature range of 30 to 160 °C; all samples were measured at a speed of 2 °C/min. The CTE of XAGP was tested using TMA 402F3 (NETZSCH, Germany) with a range of 30 to 160 °C at a speed of 10 °C/min.

NMR spectra were recorded using a Bruker AV-400 NMR spectrometer with 16 scans at room temperature. The samples (xylan, XAG) were dissolved in DMSO-d6. The degree of substitution (DS) value of XAG was calculated from their ^1^H NMR spectra. The DS and the C=C content of XAG were determined using Eqs. (1) to (3).

$$M_{\text{XAG}}=132+115.14\times \mathrm{Ds}$$ (1)

$$\text{DS}=\frac{3\times \frac{m_{\text{Br}}}{M_{\text{Br}}}\times I_{8}}{\frac{m_{\text{XAG}}}{M_{\text{XAG}}}\times I_{\text{Br}}}$$ (2)

$$C=C=\frac{1}{132+115.14\times \mathrm{Ds}}\times \text{Ds}$$ (3)

where $\;I_{8}$ is the integral area of the double bond (5.8 ppm) and $I_{\text{Br}}$ is the the integral area of BR as a reference (7.8 ppm). $m_{\text{Br}}$ and $m_{\text{XAG}}$ are the mass weight of BR and XAG, respectively.

The cytotoxicity of XAGP was assessed using the CCK-8 assay on A549 fibroblasts. Cells were exposed to XAGP at concentrations of 0 (control), 10, 50, 100, and 200 μg/ml, with cell-free medium serving as blank control. Following 24-h incubation, the medium was transferred to a 96-well plate for absorbance measurement at 450 nm. Cell viability was assessed by dual fluorescence staining using Calcein-AM (live cells, green) and propidium iodide (dead cells, red). Fluorescence images were acquired using an inverted fluorescence microscope (Olympus FV1200) with random field selection. XAGP biocompatibility was quantified according to Eq. (4).

$$\text{Cell viability}\left(\%\right)=\frac{{\text{OD}}_{\text{sample}}-{\text{OD}}_{\text{blank}}}{{\text{OD}}_{\text{control}}-{\text{OD}}_{\text{blank}}}\times 100\%$$ (4)

## Data Availability

All data needed to evaluate the conclusions in the paper are present in the paper or the Supplementary Materials.
