# Supplementary material for: Photo-Induced Synthesis of Bioplastics from Xylan
Source: Research (Wash D C). 2026 Jul 8;9:1344. doi: 10.34133/research.1344 (PMC13342247; doi:10.34133/research.1344)
Supplement: Supplementary 1 — Figs. S1 to S16 Tables S1 to S9 [file research.1344.f1.docx]

*Supporting Information*

**Photo-induced Synthesis of Bioplastics from Xylan**

Siyu Jia,^1^ Zixing Feng,^1^ Xueqing Yan,^1^ Zhiguo Zhang,^1^ Jingyan Zhu,^1^ Di Miao,^1^ Jun Rao,^1^* Zhengjun Shi,^2^ Junli Ren,^3^ Feng Peng,^1,4^*

^1^ Beijing Key Laboratory of Lignocellulosic Chemistry, MOE Engineering Research Center of Forestry Biomass Materials and Energy, School of Materials Science and Technology, Beijing Forestry University, Beijing 100083, China.

^2^ Key Laboratory for Forest Resources Conservation and Utilization in the Southwest Mountains of China, Ministry of Education, Southwest Forestry University, Kunming 650224, China.

^3^ State Key Laboratory of Pulp and Paper Engineering, South China University of Technology, Guangzhou 510641, China.

^4^ State Key Laboratory of Efficient Production of Forest Resources, Beijing 100083, China.

*Corresponding author Email address: fengpeng@bjfu.edu.cn (F. Peng), junrao@bjfu.edu.cn (J. Rao).


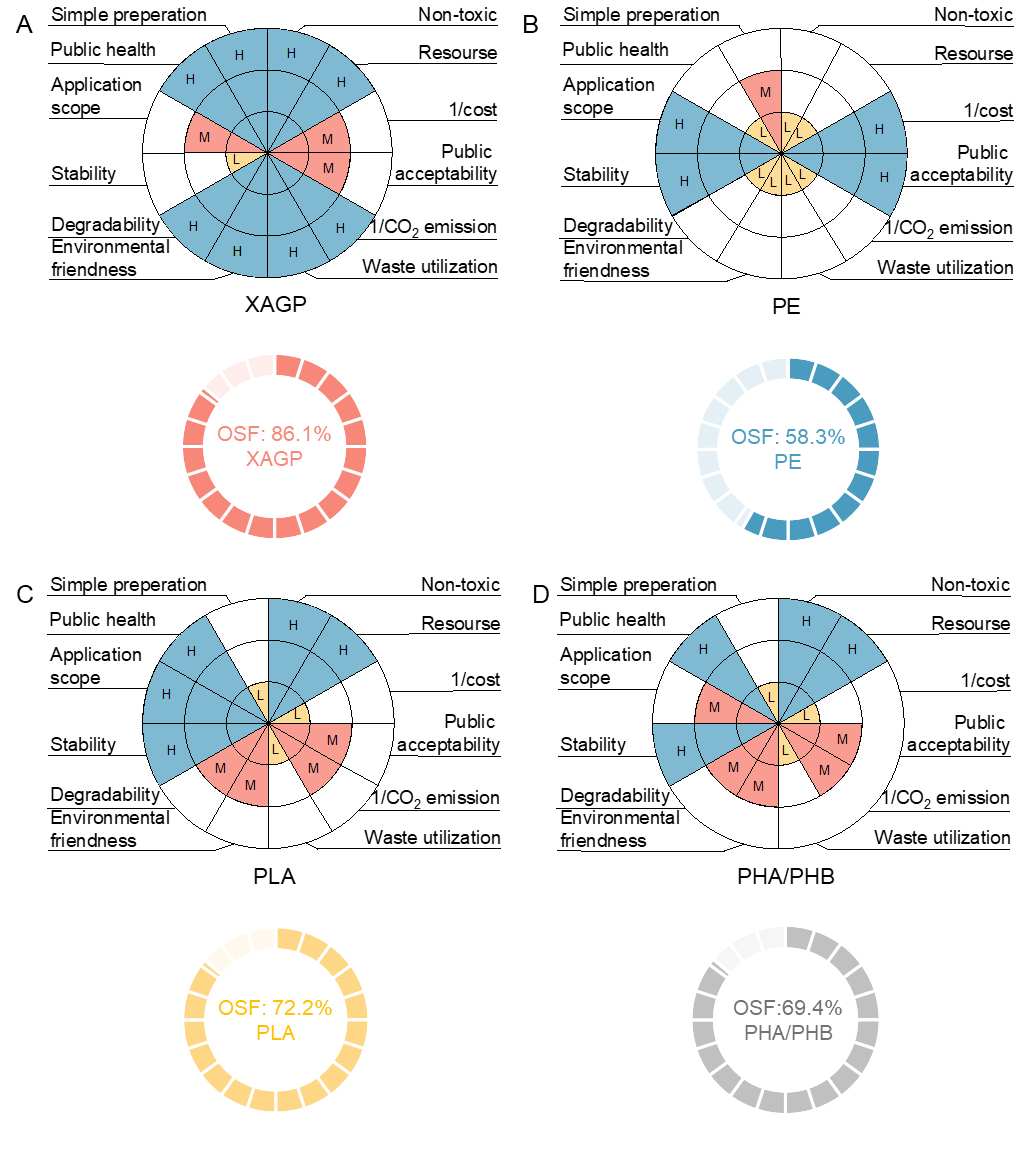


**Fig. S1** The overall sustainability footprint (OSF) of the XAGP and commercial plastic. Comparison the sustainability footprint among (A) XAGP, (B) PE plastic, (C) PLA plastic, and (D) PHA/PHB plastic. The OSF values demonstrated the good sustainability of XAGP.

With the growing concept of environmental protection, we have selected 12 aspects from environmental, social, and technoeconomic impacts to evaluate the overall sustainability footprint (OSF) of the XAGP and commercial plastic (PE, PLA, and PHA/PHB). As shown in Figure S1, the different samples were evaluated in rank order with low (L, i = 1), medium (M, i = 2), and high (H, i = 3). The XAGP exhibits significant advantages on the aspects of environmental friendliness, nature degradability, and CO_2_ emissions due to the biomass resource and easy operation. The OSF was then calculated by summing up the sustainable aspects according to the following Eq. (1).

$$OSF=100\%\times\sum_{i=1}^{12} (\frac{i}{3})i\times\frac{1}{12} (1)$$

where i represents the sustainability aspect, and i indicates the corresponding ranking score.

We obtained the OSF values of 86.1%, 69.4%, 72.2% and 58.3% for XAGP, PE plastic, PLA plastic, and PHA/PHB plastic, respectively, demonstrating the better sustainability profile of XAGP than that of synthetic plastic.


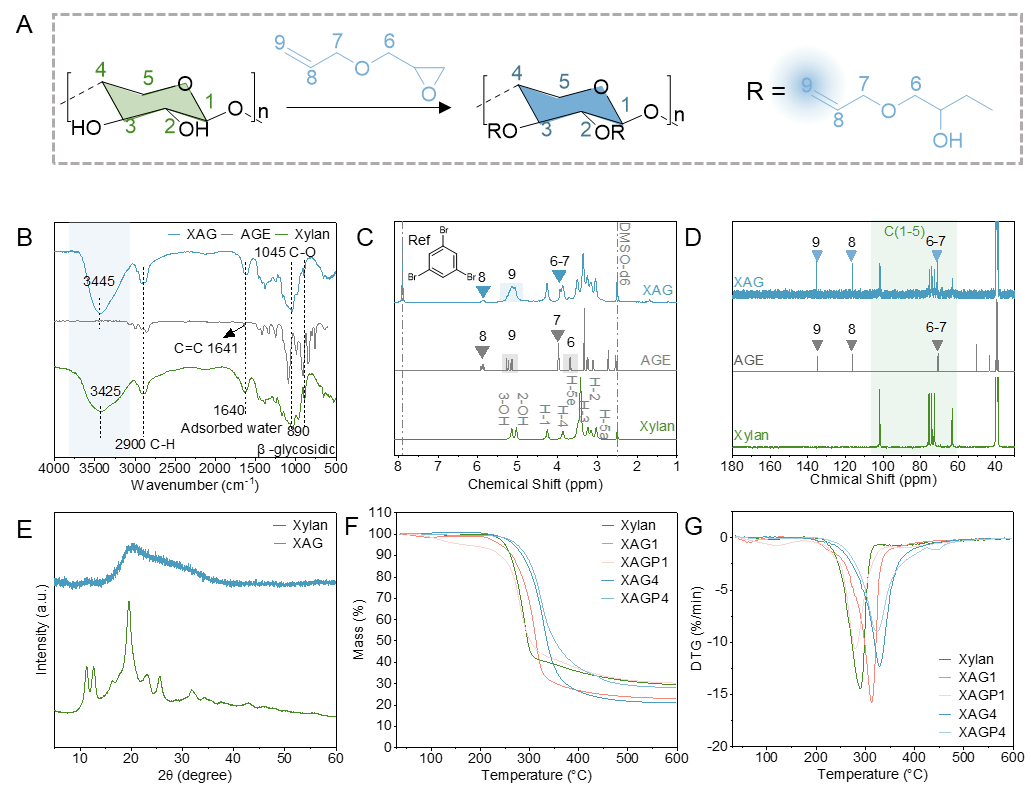


**Fig. S2** Chemical structure characterization of XAG. **(**A) Synthesis of XAG. (B) FTIR spectra, (C) ^1^H NMR spectra, and (D) ^13^C NMR spectra of xylan, AGE, and XAG. (E) XRD spectra of xylan and XAG. (F) TG and (G) DTG curves of xylan, XAG1, XAGP1, XAG4 and XAGP4.


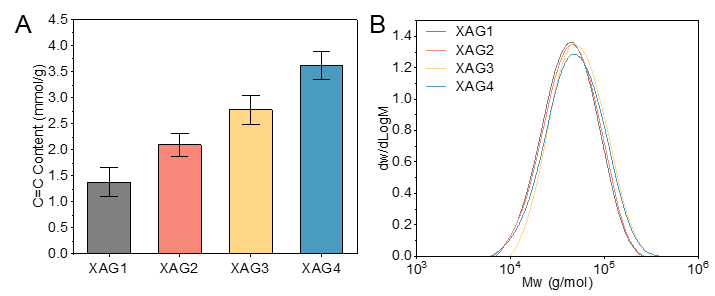


**Fig. S3** Double bond content and molecular weight of XAG at different ratios. (A) The double bond content of XAG with different ratio. (B) The molecular weight of XAG with different ratio.


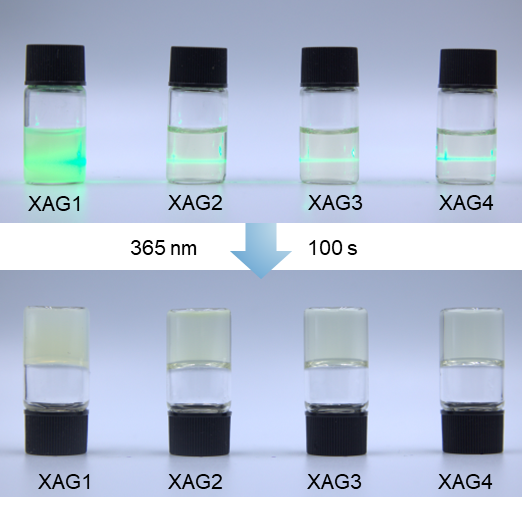


**Fig. S4** The rapid transformation of transparent XAG/I2959 solution to into a transparent XAG gel.

**
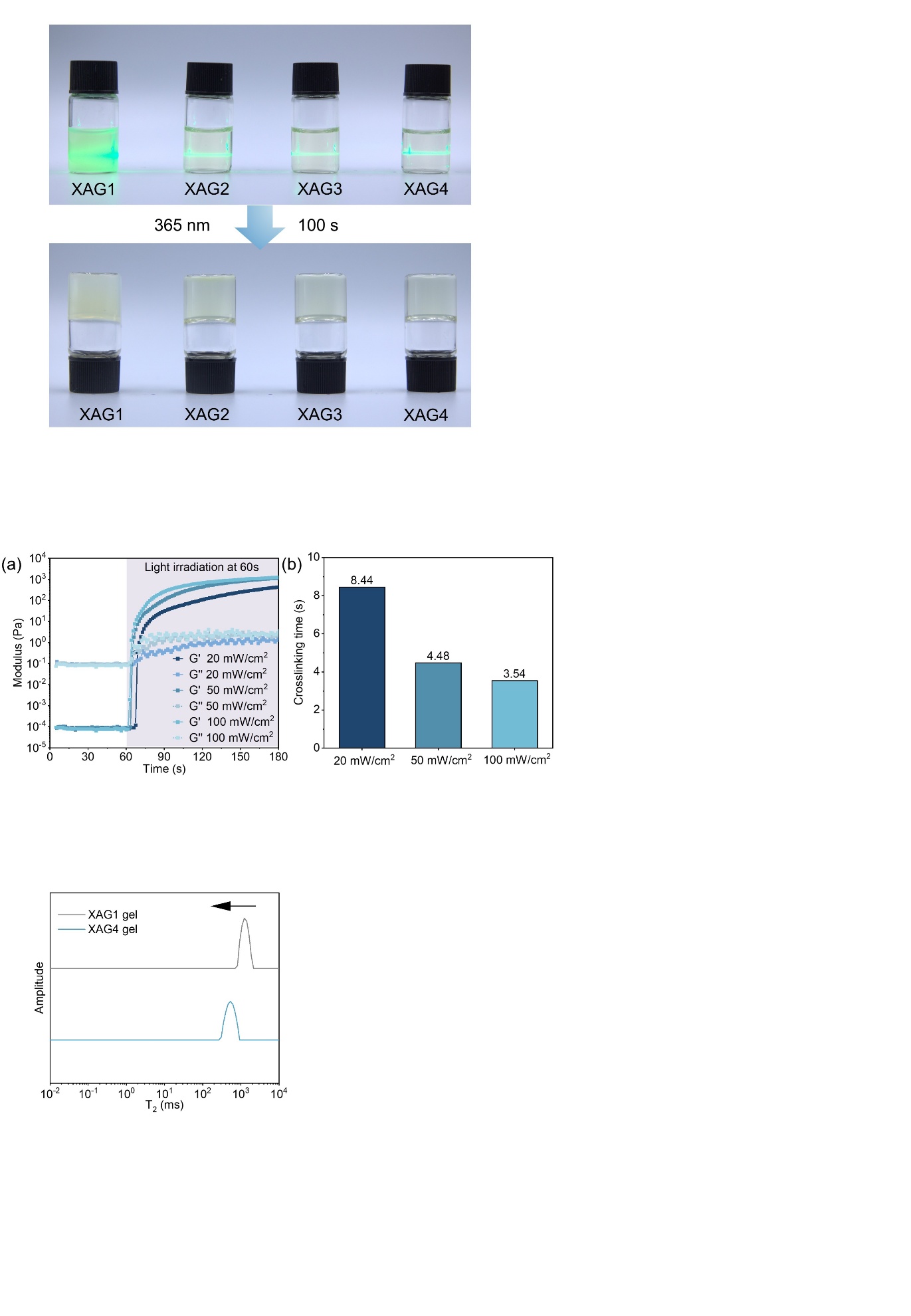
**

**Fig. S5** Compare the crosslinking degrees of different proportions of XAG gel. LF ^1^H NMR images of XAG1 gel and XAG4 gel.

**
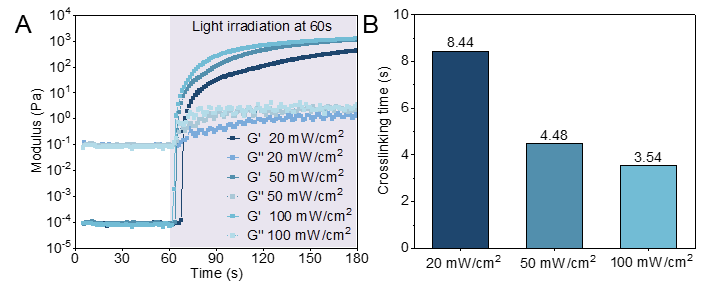
**

**Fig. S6** Crosslinking rate under different UV-light intensities. (A) Rheological monitoring and (B) gelation time of photo-crosslinking in XAG4 at different light intensity.

**
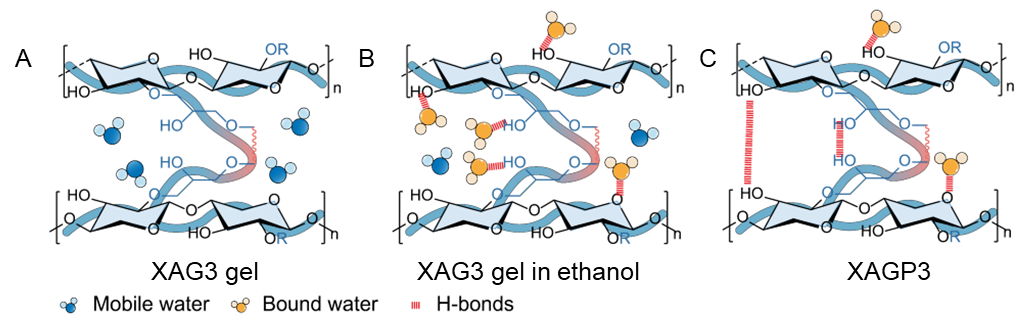
**

**Fig. S7** The internal water molecule binding situation during the preparation of XAGP. The T_2_ spectroscopy relaxation spectra from 2D-LF 1H NMR and schematic diagram of (A) XAG3 gel, (B) XAG3 gel in ethanol and (C) XAGP3.

**
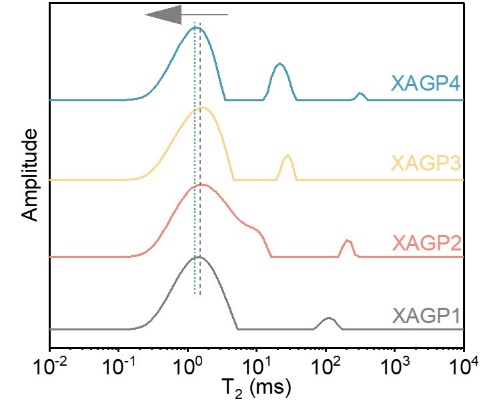
**

**Fig. S8** Compare the crosslinking degrees of XAGP with different ratio. The T_2_ spectroscopy of XAGP.


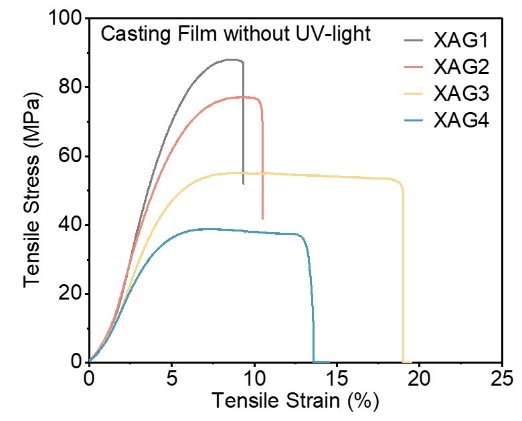


**Fig. S9** The mechanical properties of XAG casting films. Stress–strain curves of XAG casting films.

**
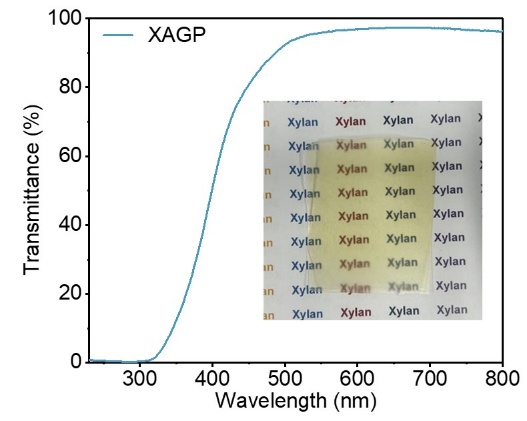
**

**Fig S10** The transparency of XAGP. The transmittance of the XAGP3.


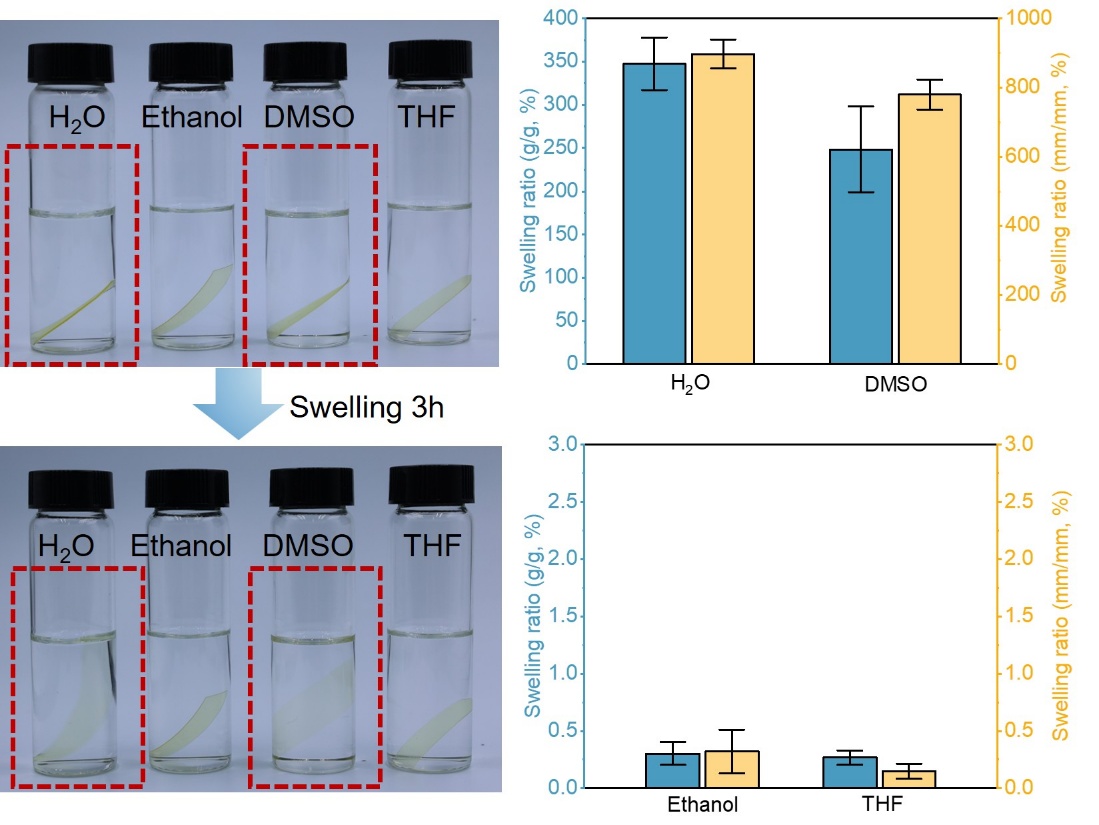


**Fig. S11** The swelling degree of XAGP. The rate of swelling in water during 3 h in water, ethanol, DMSO, and THF.


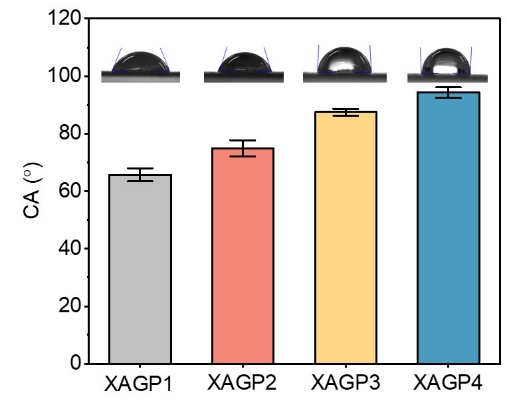


**Fig. S12** The hydrophobicity of XAGP. The water contact angle of XAGP.

**
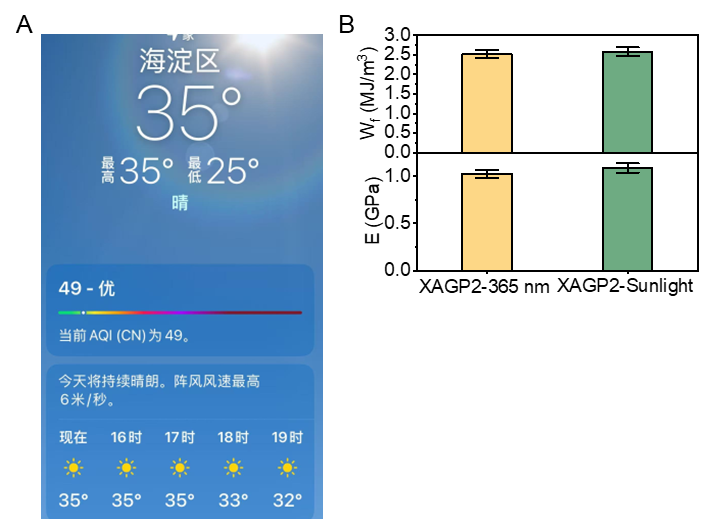
**

**Fig. S13** A Toughness (W_f_) and Young's modulus values comparing XAGP3-365 nm with XAGP3-sunlight.

**
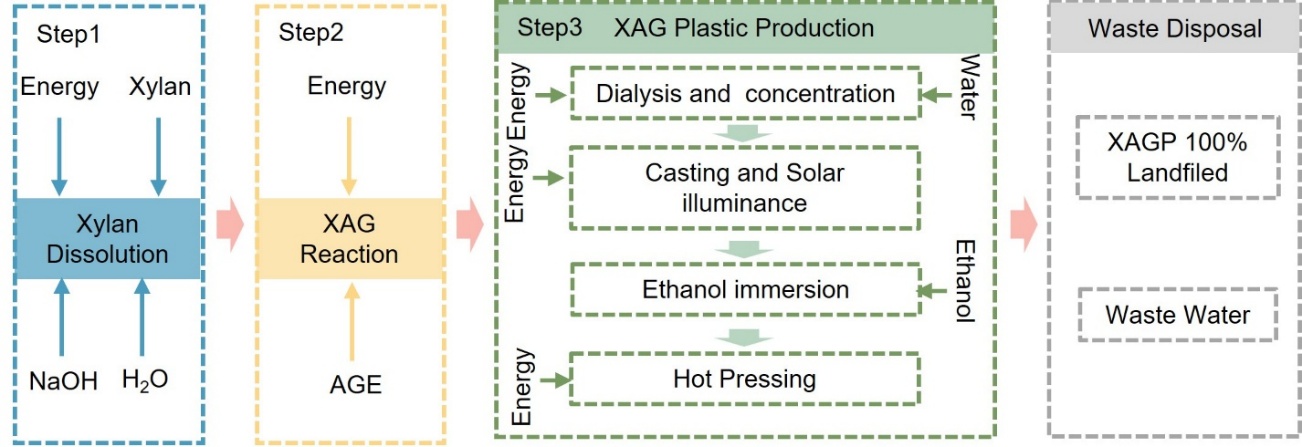
**

**Fig. S14** Material flow analysis of XAGP production.

**
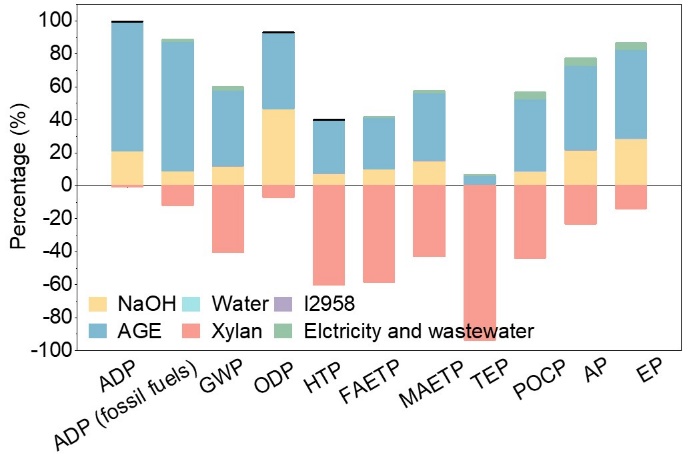
**

**Fig. S15** LCA results for XAGP.

**
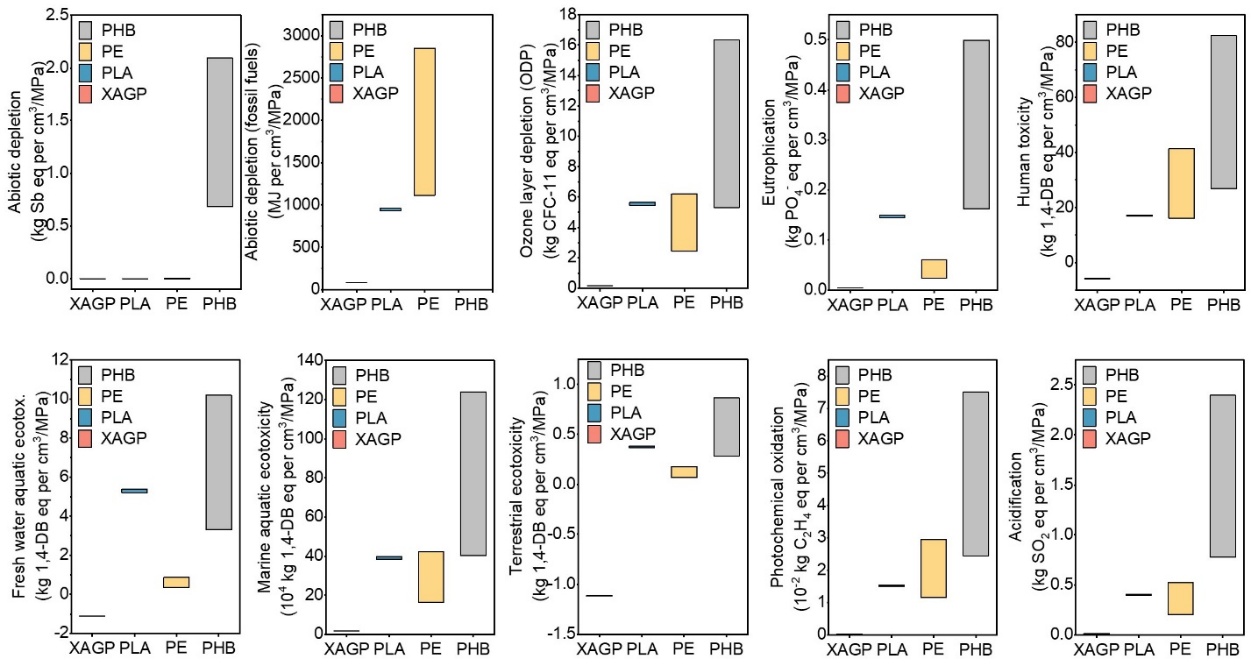
**

**Fig. S1****6** Environmental impact comparison. Environmental impact of XAGP compared to PLA, PE and PHB plastic (per cm^3^/MPa).

**Table S1** The information of XAG with different ratio.

|  | DS | C=C (mmol/g) | Mw (g/mol) | Mn (g/mol) |
| --- | --- | --- | --- | --- |
| XAG1 | 0.21±0.0734 | 1.38±0.28 | 52200 | 35007 |
| XAG2 | 0.36±0.0560 | 2.10±0.12 | 58573 | 35834 |
| XAG3 | 0.53±0.0636 | 2.77±0.26 | 63650 | 43081 |
| XAG4 | 0.82±0.0220 | 3.63±0.17 | 65514 | 45456 |

**Table S2** Comparison of mechanical properties.

| Sample | Tensile Stress (MPa) | Tensile Strain (%) | Reference |
| --- | --- | --- | --- |
| This work | 84 | 52 |  |
| Cellulose Acetate (CA) | 26 | 25 | [1] |
| Polyethylene (PE) | 24 | 2.5 |  |
| Polypropylene (PP) | 23 | 12 |  |
| Polyphenylene Sulfide (PPS) | 75 | 11 |  |
| Polyhydroxybutyrate (PHB) | 30 | 7.5 |  |
| Polylactic Acid (PLA) | 50 | 6 |  |
| Poly(butylene adipate-terephthalate)(PBAT) | 35 | 80 | [2] |
| Xylan based plastic | 55 | 50 | [3] |
| Dialdehyde starch plastic | 44 | 12 | [4] |
| Keratin plastic | 20 | 13 | [5] |
| cellulosic paper plastics | 58.2 | 15 | [6] |
| Soybean plastic | 40.82 | 23 | [7] |
| whole corn bioplastic | 78.5 MPa | 17.2 | [8] |
| konjac glucomannan bioplastic | 7.10 | 49.9 | [9] |
| Fruit peel waste plastic | 50.5 | 15 | [10] |

**Table S3** Comparison of the degradability of XAGP with other biodegradable plastics in soil.

| Type | Conditions | Temperature | Period | Biodegradability | Reference |
| --- | --- | --- | --- | --- | --- |
| Polyethylene (PE) | Nature soil,15 cm depth |  | 365 | 0 % | - |
| Poly(3-hydroxybutyrate)  (PHB) | Home composting (ISO 14855) | 28 °C | - |  | [11] |
|  | Anaerobic digestion (ISO 15985) | 52 °C | 127 d | 92% |  |
|  | Industrial composting (ISO 14855) | 58 °C | 45 d | 112% |  |
| Poly(lactic acid)  (PLA) | Home composting (ISO 14855) | 28 °C | - | - | [11] |
|  | Anaerobic digestion (ISO 15985) | 52 °C | 85 | 89% |  |
|  | Industrial composting (ISO 14855) | 58 °C | 75 | 100% |  |
| XAGP | Nature soil,15 cm depth |  | 10 d | 100 % | This work |

**Table S4** Price estimation of materials required for the production of XAG plastic by sunlight method.

|  | Raw materials | Purchase link | Price (CNY/t) | Dosage (t) | Cost (CNY/t) | Cost (US$/t) |
| --- | --- | --- | --- | --- | --- | --- |
| Material Cost | Xylan | Yibin Yatai Biological Technology [12] | 3000 | 0.66 | 1980 | 275.91 |
|  | AGE | https://www.100ppi.com/mprice/detail-14954304.html | 25000 | 1.01 | 25250 | 3,518.58 |
|  | NaOH | https://detail.1688.com/offer/545845153493.html?spm=a261y.7663282.3002526303362591.2.31c5184868mpDb&sk=order | 6000 | 0.5 | 3000 | 418.05 |
|  | I2958 | https://detail.1688.com/offer/694992921002.html?spm=a261y.7663282.351369130448463.1.289b4470Dun0DJ&sk=consign | 1000 | 0.02 | 20 | 139.35 |
|  | Ethanol | https://detail.1688.com/offer/933928436772.html?spm=a26352.13672862.offerlist.5.1af21e623R23Yc | 5584 | 0.5 | 2792 | 389.06 |
| Operating Cost | Water and Electricity | Local market | 6 /t  0.5 /kWh | - | 6500 | 905.77 |
| All-in cost | | 39542 (CNY/t) | | 5510.16 (US$/t) | | |

**Table S5** Production cost (US$) of XAGP compared to PE, PLA and PHA/PHB (1t product).

|  | Purchase link | Price (CNY/t) | Price (US$/t) |
| --- | --- | --- | --- |
| PE | https://www.plasway.com/shop/dgth88/price/purchase/0E2A16F8AD3E3804ECB652106A537D72 | 23000 | 3,205.04 |
| PLA | https://www.plasway.com/shop/jufengde/price/purchase/7E8331D4BBBBFBA44D6A310712D7C9CE | 265000 | 36986.89 |
| PHB/PHA | https://detail.1688.com/offer/722404419414.html?spm=a261y.7663282.351369130448463.1.7a57646eIC6fHe&sk=consign | 200000 | 27921.65 |
| XAGP | - | 39542 | 5510.16 |

**Table S6** The LCA list for the production of XAGP.

| Step 1 The fabrication process of Xylan/NaOH solution. | | The preparation of an aqueous solution containing 5 wt% NaOH, 6.6 wt% Xylan, and 88.4 wt% water. The mixture was heated at 50°C for 30 minutes to ensure complete dissolution of xylan. | | |
| --- | --- | --- | --- | --- |
| Item | | Number | Units | Additional data and comments |
| Input | | | | |
| NaOH | | 0.5 | kg |  |
| Xylan | | 0.66 | kg |  |
| Water | | 10 | kg |  |
| Energy | | 0.2 | kWh | The electricity is used for heating and stirring. |
| Output | | | | |
| Xylan/NaOH solution | | 11.16 | kg |  |
| Step 2: The fabrication Process of XAG2. | | The AGE was added dropwise to the Xylan/NaOH solution and stirred for 24 h. | | |
| Item | | Number | Units | Additional data and comments |
| Input | | | | |
| Xylan/NaOH solution | | 11.16 | kg |  |
| AGE | | 1.01 | kg |  |
| Energy | | 0.2 | kWh | The electricity is used for stirring. |
| Output | | | | |
| XAG solution | | 12.17 | kg |  |
| Step 3: The production process of XAG2 plastic. | |  | | |
| Item | | Number | Units | Additional data and comments |
| Input | | | | |
| Dialysis | Water | 10 | kg |  |
| Concentration | Energy | 20 | kWh | The electricity is used for concentration. |
| I2958 | | 0.02 | kg |  |
| Casting | Energy | 0.1 | kWh | The electricity is used for transmission. |
| Solar illuminance | | 0 |  |  |
| Ethanol immersion | | 50 | kg | Since ethanol can be recycled 100% and is not included in the LCA calculation. |
| Hot Pressing | Energy | 0.8 | kWh | The electricity is used for hot pressing. |
| Output | | | | |
| XAG Plastic | | 1 | kg |  |
| COD | | 2.88 | kg |  |
| BOD5 | | 5 | kg |  |
| Waste water | | 8 | kg |  |

**Table S7** Comparison of LCA characterization data between XAGP and PE/PLA/PHB (1t functional unit).

| Impact category |  | Unit | XAGP | PE | PLA | PHB  [13] |
| --- | --- | --- | --- | --- | --- | --- |
| Abiotic depletion | ADP | kg Sb eq | 2.94E-03 | 0.012098 | 0.008368 | 2.180E+01 |
| Abiotic depletion (fossil fuels) | ADP  (fossil fuels) | MJ | 6.87E+03 | 68468.51 | 37562.08 | - |
| Global warming (GWP100a) | GWP | kg CO_2_ eq | 1.19E+02 | 3164.071 | 3238.761 | 1.960E+03 |
| Ozone layer depletion (ODP) | ODP | kg CFC^-11^ eq | 1.36E-05 | 0.000149 | 0.00022 | 1.700E-04 |
| Human toxicity | HTP | kg 1,4-DB eq | -4.77E+02 | 991.0334 | 676.9023 | 8.570E+02 |
| Fresh water aquatic ecotox. | FAETP | kg 1,4-DB eq | -9.08E+01 | 20.95358 | 209.8052 | 1.060E+02 |
| Marine aquatic ecotoxicity | MAETP | kg 1,4-DB eq | 1.46E+05 | 1010087 | 1550547 | 1.290E+06 |
| Terrestrial ecotoxicity | TEP | kg 1,4-DB eq | -9.14E+01 | 4.207257 | 14.79053 | 8.980E+00 |
| Photochemical oxidation | POCP | kg C_2_H_4_ eq | 2.00E-02 | 0.707596 | 0.600265 | 7.800E-01 |
| Acidification | AP | kg SO_2_ eq | 8.32E-01 | 12.50196 | 15.93613 | 2.490E+01 |
| Eutrophication | EP | kg PO_4_^-^eq | 3.79E-01 | 1.469533 | 5.846473 | 5.190E+00 |

**Table S8** Density and tensile strength of PE, PLA, PHB and XAGP.

|  | Density (g/cm^3^) | Tensile strength (MPa) | Ref. |
| --- | --- | --- | --- |
| PE | 1.0-1.3 | 24-80 | https://www.makeitfrom.com/ |
| PLA | 1.24-1.28 | 50 | https://www.makeitfrom.com/ |
| PHB | 1.25 | 13-40 | [13] |
| XAGP | 0.97-1.02 | 83.8-79.2 | This work |

**Table S9** Environmental impact of XAGP compared to PE, PLA, and PHB (per cm3/MPa).

| Impact category |  | Unit | XAGP | PE | PLA | PHB |
| --- | --- | --- | --- | --- | --- | --- |
| Abiotic depletion | ADP | kg Sb eq | 3.60E-05 | 5.04E-04 | 0.000207526 | 2.10E+00 |
| Abiotic depletion (fossil fuels) | ADP  (fossil fuels) | MJ | 8.41E+01 | 2.85E+03 | 931.539584 | #VALUE! |
| Global warming (GWP100a) | GWP | kg CO_2_ eq | 1.46E+00 | 1.32E+02 | 80.3212728 | 1.88E+02 |
| Ozone layer depletion (ODP) | ODP | kg CFC^-11^ eq | 1.67E-07 | 6.19E-06 | 0.000005456 | 1.63E-05 |
| Human toxicity | HTP | kg 1,4-DB eq | -5.84E+00 | 4.13E+01 | 16.78717704 | 8.24E+01 |
| Fresh water aquatic ecotox. | FAETP | kg 1,4-DB eq | -1.11E+00 | 8.73E-01 | 5.20316896 | 1.02E+01 |
| Marine aquatic ecotoxicity | MAETP | kg 1,4-DB eq | 1.79E+03 | 4.21E+04 | 38453.5656 | 1.24E+05 |
| Terrestrial ecotoxicity | TEP | kg 1,4-DB eq | -1.12E+00 | 1.75E-01 | 0.366805144 | 8.63E-01 |
| Photochemical oxidation | POCP | kg C_2_H_4_ eq | 2.45E-04 | 2.95E-02 | 0.014886572 | 7.50E-02 |
| Acidification | AP | kg SO_2_ eq | 1.02E-02 | 5.21E-01 | 0.395216024 | 2.39E+00 |
| Eutrophication | EP | kg PO_4_^-^eq | 4.64E-03 | 6.12E-02 | 0.14499253 | 4.99E-01 |

**References**

[1] Material properties database. https://www.makeitfrom.com.

[2] Jiang, B., Wang, Y., Peng, Z., Lim, K. H., Wang, Q., Shi, S., Zheng, J., Yang, X., Liu, P., Wang, W.-J., Synthesis of poly(butylene adipate terephthalate)-co-poly(glycolic acid) with enhanced degradability in water. *Macromolecules* 2023;56(22):9207-9217.

[3] Jia, S., Lv, Z., Rao, J., Lü, B., Chen, G., Bian, J., Li, M., Peng, F., Xylan plastic. *ACS Nano* 2023;17(14):13627-13637.

[4] Ding, Y., Liu, D., Sun, Y., Liu, S., Wang, P., Wang, S., Huang, D., Ji, J., Dynamic imine bond-enabled starch-based materials with self-healing and recycling properties. *ACS Sustainable Chem. Eng.* 2025;13(17):6388-6398.

[5] Trojanowska, D. J., Zych, A., Sganga, S., Tirelli, N., Boventi, M., Rinaldi, C., Simonutti, R., Athanassiou, A., Perotto, G., Upgrading keratin into a moldable bioplastic. *Matter* 2025;8(4):102039.

[6] Li, X., Li, X., Ma, W., Ma, J., An in-situ dissolving-co-crosslinking strategy for fabricating high-strength, wet-stable, and biocompatible multiscale cellulosic paper-based plastics. *Carbohydr. Polym.* 2025;355:123347.

[7] Zhang, X., Li, P., Zeng, J., Su, J., Xu, J., Li, J., Wang, B., Gao, W., Chen, K., Dynamically crosslinking cellulose nanofibers and epoxy soybean oil toward tough, recyclable, and degradable bioplastics. *ACS Sustainable Chem. Eng.* 2024;12(50):18174-18186.

[8] Xie, D., Yang, S., Zhang, C., Zhang, R., Yang, A., Zhu, Y., Xia, Q., Wang, H., Song, S., Song, Y., A robust, recyclable, and biodegradable whole corn bioplastic enabled by dissolution-regeneration strategy. *Chem. Eng. J.* 2024;501:157571.

[9] Zhou, H., Zhang, X., Zhou, G., Demi̇r, M., Lei, Z., Zhang, W., Wang, X., Water-mediated synthesis of full-biomass vitrimer with enhanced moldability, recyclability, and biodegradability. *ACS Sustainable Chem. Eng.* 2024;12(18):6952-6959.

[10] Zhang, S., Li, H., Zhang, B., Ai, S., Shan, Y., Ding, S., Non-covalent in situ self-assembly of fruit peel waste into eco-friendly pectocellulosic bioplastics with high strength, flexibility and processability properties. *Chem. Eng. J.* 2025;504:158697.

[11] Narancic, T., Verstichel, S., Reddy Chaganti, S., Morales-Gamez, L., Kenny, S. T., De Wilde, B., Babu Padamati, R., O’Connor, K. E., Biodegradable plastic blends create new possibilities for end-of-life management of plastics but they are not a panacea for plastic pollution. *Environ. Sci. Technol.* 2018;52(18):10441-10452.

[12] Lv, Z., Yan, X., Jia, S., Pan, J., Hao, X., Chen, G., Lü, B., Rao, J., Peng, F., Bio-based hot-melt adhesive from xylan. *Nat. Sustain.* 2025;8(7):827-836.

[13] Zhou, H., Mao, Y., Zheng, Y., Liu, T., Yang, Y., Si, C., Wang, L., Dai, L., Complete conversion of xylose-extracted corncob residues to bioplastic in a green and low carbon footprint way. *Chem. Eng. J.* 2023;471:144572.
